# The Role of Maternal Vitamin D Deficiency in Offspring Obesity: A Narrative Review

**DOI:** 10.3390/nu15030533

**Published:** 2023-01-19

**Authors:** Yifan Wu, Yuan Zeng, Qian Zhang, Xinhua Xiao

**Affiliations:** 1 Key Laboratory of Endocrinology, Ministry of Health, Department of Endocrinology, Peking Union Medical College Hospital, Peking Union Medical College, Chinese Academy of Medical Sciences, Beijing 100730, China; 2 State Key Laboratory of Complex Severe and Rare Diseases, The Translational Medicine Center of Peking Union Medical College Hospital, Chinese Academy of Medical Sciences, Beijing 100730, China

**Keywords:** vitamin D deficiency, obesity, Developmental Origins of Health and Disease (DOHaD), offspring, pregnancy

## Abstract

Currently, vitamin D (VD) deficiency during pregnancy is widespread globally, causing unfavorable pregnancy outcomes for both mothers and infants for a longer time than expected, based on the Developmental Origins of Health and Disease (DOHaD) theory. As VD plays a key role in maintaining normal glucose and lipid metabolism, maternal VD deficiency may lead to obesity and other obesity-related diseases among offspring later in life. This review mainly focuses on the effect of maternal VD deficiency on offspring lipid metabolism, reviewing previous clinical and animal studies to determine the effects of maternal VD deficit on offspring obesity and potential mechanisms involved in the progression of offspring obesity. Emerging clinical evidence shows that a low VD level may lead to abnormal growth (either growth restriction or largeness for gestational age) and lipid and glucose metabolism disorders in offspring. Here, we also outline the link between maternal VD deficiency and life-long offspring effects, including the disorder of adipogenesis, the secretion of adipocytokines (including leptin, resistin, and adiponectin), activated systemic inflammation, increased oxidative reactions in adipose tissue, insulin resistance, and abnormal intestinal gut microbiota. Thus, there is an urgent need to take active steps to address maternal VD deficiency to relieve the global burden of obesity.

## 1. Introduction

Overweight and obesity have become public health problems worldwide. Published in 2018, data from the World Health Organization (WHO) showed that more than 1.9 billion adults (≥ 18 years old) were overweight and over 650 million were obese in 2016, which is nearly triple the number in 1975 [1]. Obesity poses a high risk for a vast number of serious chronic noncommunicable diseases, including type 2 diabetes, cardiovascular diseases, and cancers [2,3].

Currently, the mechanism of obesity is not fully understood. It is generally acknowledged that the interaction of genetic and environmental factors contributes to the occurrence of obesity [4,5], coupled with hypertrophy and/or hyperplasia of adipose tissue [6], disorders in glucose and lipid metabolism [7], and increased inflammatory responses within adipose tissue [8]. Furthermore, the perception “Developmental Origins of Health and Disease” (DOHaD), evolved from the “thrifty phenotype” explanation created by Barker in 1992, claimed that it is in the very early life, the stage of gestation, during which unfavorable environmental exposure can robustly pose a high incidence of chronic metabolism disorders, such as diabetes, obesity, dyslipidemia, etc., which persist in offspring into adulthood and even through generations [9,10,11]. Such an effect is brought about through epigenetic mechanisms, including DNA methylation, histone modifications, and noncoding RNAs [12], by altered intrauterine environment factors, including maternal nutrition status [13], lack of physical activities [13,14], circadian dysregulation [15], psychological stress [16], and tobacco smoking [17] during gestation.

Vitamin D (VD) is both a fat-soluble vitamin and a steroid hormone that is mainly synthesized endogenously through skin exposure to ultraviolet B (UVB) [18]. VD is an essential regulator of phosphate and calcium homeostasis and plays a key role in bone metabolism [19]. Additionally, VD has many extraskeletal benefits and its deficiency has been reported to be related to a wide range of diseases, such as asthma [20], cancers [21], type 2 diabetes [22], cardiovascular diseases [23], infections [24,25], and autoimmune diseases [25,26]. A systematic review stated that VD level is inversely correlated with the percentage of fat mass [27]. In addition, based on the DOHaD theory, maternal VD deficiency also affects epigenetic modifications [28], and therefore makes offspring more vulnerable to obesity later in life. Here, we review the current knowledge about the effect of maternal VD deficiency on obese offspring with adipose tissue dysfunction.

## 2. The Relationship of VD Deficiency during Pregnancy and Obesity-Related Diseases in Offspring

### 2.1. VD Metabolism

VD is a lipid-soluble steroid and has two main forms of supplementation, ergocalciferol (vitamin D_2_, naturally produced by fungi organisms in UVB radiation and often prescribed in the U.S. [29]) and cholecalciferol (vitamin D_3_, mainly produced by skin in response to sunlight and obtained from D_3_-enriched or fortified food, another important source of D_3_ [30], such as fish, meat, eggs, etc. [31]). The binding of vitamin D_2_ metabolites to vitamin D binding protein (DBP) in plasma is diminished compared with vitamin D_3_ [32]. Additionally, it has been reported that vitamin D_3_ is more potent and has a longer-lasting effect than vitamin D_2_ [33]. Therefore, for patients in the need of VD supplementation, vitamin D_3_ should be selected over vitamin D_2_.

VD is mainly obtained from skin synthesis through sun exposure, food intake (oily fish, eggs, red meat, etc.), and VD supplementation. First, VD is ingested or produced in the skin, which results from the UVB (290~315 nm)-mediated photolytic-conversion of 7-dehydrocholesterol (DHC) in skin. Then, it binds to DBP and is hydroxylated to 25-hydroxyvitamin D [25(OH)D] in the liver. Finally, 25(OH)D was further hydroxylated primarily in the kidney to the physiologically active 1,25-dihydroxyvitamin D (1,25(OH)_2_D), catalyzed by 1α-hydroxylase. Additionally, a previous study reported that 1α-hydroxylase was detected in extrarenal organs and tissues, including skin, lymph nodes, colon, pancreas, adrenal medulla, brain, and placenta [34], suggesting a potential effect of VD in these organs and tissues. In this process, adipose tissue functions as a major reservoir for VD, pooling 73% of the cholecalciferol and 34% of 25(OH)D in the body. However, the mechanism of VD uptake and release in adipose tissue is still unclear, but evidence demonstrates that many mechanisms, including the megalin/cubilin pathway, cholesterol (TC) transporters, and other mechanisms, are involved in 25(OH)D uptake. In addition, lipolysis may be involved in VD release, and this process is reported to be blunted in obese individuals, suggesting that the adipose is a pool of VD and that the release of poor VD from adipose tissue may cause lower serum VD levels in obese people [35]. This suggests that obesity is a contributing factor to VD deficiency independent of insufficient VD intake and lack of sunshine.

### 2.2. VD Biological Effects

After absorption or synthesis through skin, VD is hydroxylated to its active form, 1,25(OH)_2_D, and interacts with vitamin D receptor (VDR), a nuclear transcription factor. Apart from classical target organ cells, such as the intestine, parathyroid gland, and bone, VDR is also widely present in tissues, such as the heart, skin, brain, pancreas, and immune system, and moderates certain biologic effects, including glucose and lipid metabolism, cell proliferation and differentiation, and the activation of immune cells. In obese individuals, VDR expression is elevated in adipose tissue compared with lean individuals [35]. Additionally, uncoupling protein-1 (UCP-1) is a member of the uncoupling protein family involved in thermogenesis, energy metabolism, and obesity. The absence of UCP-1 augmented obesity in high-fat and cafeteria-fed mice [36]. In an animal study, VDR knockout C57BL6 mice resulted in UCP-1 expression and protection from diet-induced obesity, which means disrupted expression of VDR results in a compensatory increase in UCP-1 expression [37]. This indicates that the degree of obesity is associated with the response of adipose tissue to VD via VDR and VDR is involved in the alternations in adipose tissue during the progression of obesity.

The genomic effects activate the transcription of VD-related genes through the interaction between VD and VDR which heterodimerizes with retinoid X receptor (RXR) and binds to Vitamin D response elements (VDRE) in the promoter regions after the dimer translocates to the nucleus [18]. VD controls the transcription of hundreds of genes in a species- and cell-specific manner, such as nuclear factor kappa B (NF-κB), CCAAT enhancer binding proteins (C/EBP), and signal transducer and activator of transcription 5 (STAT5) [38,39,40].

VD is engaged in several signaling pathways, referred to as nongenomic effects of VD. Once VD is bound to the membrane VDR located in the plasma membrane, the membrane VDR interacts with proteins, including phospholipases C and A2, phosphoinositide 3-kinase, and calcium transporters. Then, it activates the secondary messengers and its downstream protein kinases A and C, mitogen-activated protein kinases (MAPKs), and Ca^2+^-calmodulin kinase II. These rapid and short-lasting nongenomic effects of VD also participate in the regulation of gene expression by influencing the transcription of genes [41].

Regarding epigenetic effects, several animal models have revealed that VD moderates the progression of DNA methylation [42,43,44]. Moreover, VD is involved in histone acetylation, including the activation of histone acetyltransferases (HATs) and histone deacetylases, as well as histone methylation and demethylation [45]. Furthermore, VD plays a role in the regulation of microRNAs (miRNAs), attenuating inflammation by limiting inflammation-related miRNA expression [46]. VDR and its ligand VD belong to the factors influencing the activity of chromatin modifiers and thus are modulators of the human epigenome. Acetylation is associated with the interaction between the VDR/RXR dimer and HATs. However, other epigenetic mechanisms remain scarce [47,48].

In mitochondria, VDR is reported to have a negative effect on mitochondrial respiratory capacity in liver, muscle, adipocytes, and platelets [49,50,51]. In muscle, VDR is a key intermediate between VD status and mitochondrial function [52]. The ablation of VDR enhances mitochondrial respiratory activity and the production of reactive oxygen species (ROS), triggering long-term cellular damage and cell death [49]. This suggests that VD and VDR are crucial for mitochondrial oxidative phosphorylation capacity, playing an antioxidant role in protecting cells from ROS. Specifically, VDR affects the progression of lipid biosynthesis by regulating the tricarboxylic acid cycle (TCA cycle). In muscle, when VDR is ablated, the increased respiratory activity enhances the TCA cycle and β-oxidation. Consequently, fat is mobilized from depots and catabolized in mitochondria, leading to muscle waste [53].

### 2.3. VD Deficiency in Pregnant Women

Low maternal VD status during pregnancy is crucial to various health outcomes in the offspring, ranging from periconceptional effects to diseases of adult onset [54]. VD deficiency is diagnosed and monitored based on the level of serum calcitriol [25(OH)D_3_], which reflects the summation of dietary VD intake and VD produced from solar UVB exposure but not the storage of VD. VD deficiency is defined as a cutoff level of 50 nmol/L (20 ng/mL) to avoid bone problems; and VD insufficiency is defined as a serum 25(OH)D of 52.5~72.5 nmol/L (21~29 ng/mL) [55,56]. However, currently, there is no consensus on an optimal level during pregnancy.

Evidence has demonstrated that VD deficiency remains prevalent among pregnant women around the world with consistent evidence suggesting an incidence of 51% to 100% in developing countries [57] and a reemergence in developed countries [58], especially those with high-risk factors, including vegetarians, limited sunlight exposure (such as living in a cold climate, frequently wearing protective clothes and sunscreen, lack of outdoor activities), malnutrition, obesity, and ethnic groups with dark skin [59]. However, data on VD status and VD deficiency were mainly outdated (over five years old) and/or involved relatively small studies of selected samples. There is still a lack of representative population-based surveys on the prevalence of VD deficiency among pregnant women.

Cord VD level is strongly linked with maternal VD status, especially that in the third trimester, as reported in a systematic review and meta-analysis (pooled r = 0.8, *p* < 0.001 during the third trimester vs. pooled r = 0.4, *p* = 0.01 before the third trimester) [60]. A RCT of VD supplementation in pregnancy (4400 IU/day vs. 400 IU/day) found that every 1 ng/mL increase in third trimester maternal 25(OH)D was associated with an increase in cord 25(OH)D of 0.43 ng/mL (1.1 nmol/L) (SE 0.05, *p* < 0.001) in the 4,400 IU/day group and of 0.30 ng/mL (0.8 nmol/L) (SE 0.06, *p* < 0.001) in the 400 IU/day group [61]. Newborns with VD deficiency was also associated with maternal VD deficient (OR = 6.9, 95 % CI 3.1–15.4, *p* < 0.01) [62]. Consequently, infants whose mothers are with or at high risk of VD deficiency are also at risk of VD deficiency [63]. All the findings indicate the importance of monitoring maternal VD concentrations regularly in every phase of pregnancy, including late pregnancy. Additionally, VD deficiency during pregnancy is related to many pregnancy complications and adverse outcomes, including preeclampsia, gestational diabetes mellitus (GDM), bacterial vaginosis, preterm birth, adverse neurodevelopmental outcomes, and underdeveloped fetuses, such as small for gestational age [64,65,66,67,68,69]. There is also evidence of the high VD concertation (VD > 75 nmol/L) during pregnancy increasing the risk of eczema and asthma at nine years of age, which needs more evidence to confirm [70].

The optimal and safe dose to correct VD deficiency in pregnancy remains unclear and needs more data and research [71]. A previous study reported that 4000 IU/day is safe for pregnant women and their fetus [72], and 6400 IU/day is safe for women during lactation and their infants [73]. Moreover, among all these approaches for analysis, evidence-based medicine (EBM) and randomized controlled trial (RCT), are recognized as the “gold standards” so far. However, Dr. Heaney [74] pointed out that EBM, as suitable to the study of drugs, is inappropriate when applied to the evaluation of nutrients, including VD studies, and posed standards for RCT on nutrients. A RCT that satisfied Heaney’s standards reported that 50,000 IU/week plus a maintenance dose of 50,000 IU/month during pregnancy improved the probability for achieving serum 25(OH)D levels above 20 ng/mL [75]. In all, more evidence that based on Heaney’s rules is needed to determine the optimal dose of VD for pregnant women.

On one hand, as mentioned above, due to the lack of RCTs, the Institutes of Medicine (IOM) give a conservative recommendation of 600 IU/day, no more than 4000 IU/day, for pregnant women [76]. In addition, the National Institute for Health and Care Excellence (NICE) recommends that all pregnant individuals intake 10 mcg (400 UI) per day, whether they have VD deficiency or not, and a higher dose of VD supplementation may be considered if local laboratory results indicate a need for treatment [77]. In the American College of Obstetricians and Gynecologists (ACOG) guidelines, a daily intake of 1000~2000 UI is advised among pregnant women with VD deficiency, with upper limits of 4000 UI and a daily intake of 600 UI is recommended for all pregnant women [78]. In South Australia, a daily intake VD of 400 UI is recommended for pregnant women with VD sufficiency and pregnant women with VD deficiency need to consume 1000 UI VD daily [79]. Monitoring following treatment can begin approximately six weeks after starting treatment, and the assessment of neonatal calcium and VD levels at delivery is reasonable if it is necessary [79].

On the other hand, however, existing clinical evidence has reported that higher VD dose and serum 25(OH)D concentrations were more beneficial, and further increases in recommendations for VD supplementation are needed. Based on a RCT following Heaney’s rules [74], VD supplementation of 50,000 IU/week plus maintenance dose of 50,000 IU/month helped pregnant women achieve serum 25(OH)D > 20 ng/mL during pregnancy, and, after supplementation, screening reduced the incidence of adverse pregnancy outcomes, including preeclampsia, GDM, and preterm delivery [75]. An intervention observational study reported that pregnant women with 25(OH)D ≥ 40 ng/mL had a lower risk of preterm birth (OR = 0.41, *p* = 0.002) [80]. Women with 25(OH)D < 37.5 nmol/L were more likely to have a cesarean section than those with 25(OH)D ≥ 37.5 nmol/L (OR = 3.8, 95% CI 1.7~8.6) [81]. Additionally, to achieve the high VD level of 40~60 ng/mL, a daily intake of 4000 IU vitamin D_3_ is required [82]. A study also reported that VD supplementation of 4000 IU/day for pregnant women is safe and most effective in achieving VD sufficiency and reducing preeclampsia and caesarean section, compared with a lower dose (400 or 2000 IU/day) [83,84]. Thus, The Endocrine Society recommended that pregnant and lactating women require at least 600 IU/day of VD and at least 1500~2000 IU/day of VD may be needed to maintain a blood level of 25(OH)D above 30 ng/mL [56]. Moreover, the Endocrine Practice Guidelines Committee suggested a daily requirement of 600~1000 IU for 14~18 years old and 1500~2000 IU for 19~50 years old, with upper limits of 10,000 IU/day [56].

### 2.4. Clinical Evidence of VD Deficiency in Obese Pregnant Women and Its Effect on Offspring

Obesity during pregnancy has short- and long-term adverse consequences for both the mother and child. For pregnant women, obesity increases the risk of insulin resistance in early pregnancy, leading to fetal overgrowth. Consequently, the rate of cesarean delivery and wound complications is higher among obese pregnant women. Moreover, obese women have an increased risk of future cardiometabolic [85]. Therefore, it is important to control weight gaining during pregnancy. In fact, maternal obesity during pregnancy is linked to socio-demographic, lifestyle, and genetic factors [86]. There is limited evidence to support that maternal VD deficiency will lead to obesity among pregnant women. However, VD deficiency during pregnancy predispose obese pregnant women to disorders in glucose and lipid metabolism, which also increases the risk of diabetes and obesity in later life for offspring. Obese women during pregnancy are more likely to have lower levels of VD compared to nonobese women, as has been reported by several clinical studies because of the increased storage of VD in adipose tissue [87]. In view of the adverse effect of VD deficiency during pregnancy on the metabolism of glucose and metabolism in general, obese pregnant women are more likely to be vulnerable to metabolic disorders.

For offspring, neonates of obese women tend to have higher body fat at birth, increasing the risk of childhood obesity and chronic metabolism disorders, such as type 2 diabetes, cardiovascular diseases, and dyslipidemia, etc. [85]. In an observational study, maternal VD status was associated with neonatal anthropometric measures varied by maternal adiposity status and gestational weeks, indicating that overweight/obese pregnant women may need to prevent VD deficiency, especially in early and late pregnancy to optimize fatal growth [88].

In addition, maternal VD deficiency has an obesity-independent effect on offspring obesity. Newborns of women with VD deficiency during pregnancy reportedly tend to have a higher abdominal subcutaneous adipose tissue volume within two weeks post-delivery [89], and a higher body mass index (BMI) at six years of age [90,91]. Crozier et al. [92] reported that low VD status is linked to greater fat mass among offspring at four and six years of age. Morales et al. [93] found that maternal VD deficiency is related to the risk of offspring obesity at one year old (OR = 1.4, 95% CI: 1.0–2.0; *p* = 0.04), but not at four-years-old (OR = 1.2, 95% CI: 0.8–1.8; *p* = 0.3). Jiang et al. [94] observed no association between maternal VD deficiency and adverse pregnancy outcomes, including preterm birth, small for gestation age, and low birth weight, as well as anthropometric indices (such as weight, length, BMI) at 0–3 years of age. In a rat model [95], dams were fed on a VD-free diet and compared with the group of normal diet-fed dams. The group of VD-depleted diet-fed dams showed no significant difference in body weight during pregnancy of dams and from birth to 16 weeks in the offspring, but there was a decrease in VD level at birth in the offspring. In another rat study [42], a significant increase in proliferation rate and number of lipid droplets for pre-adipocytes was observed in the offspring of the VD-restricted diet (0 UI/kg vs. 1000 UI/kg) group, suggesting that VD deficiency among pregnant women, no matter whether obese or nonobese, poses a higher threat of obesity to offspring in later life. In addition, adequate VD is optimal for fetal and child health. Since the active form of VD, calcitriol, cannot enter the placenta, the DBP-bound 25(OH)D_3_ in the plasma is absorbed by the placenta, and then hydroxylated in the placenta and fetal kidney to transform into the active form. In addition, VD is involved in regulating the development of the placenta and fetus [96]. Therefore, VD deficiency is also threatening to the development of fetal and normal placental function.

### 2.5. Clinical Evidence of VD Deficiency in GDM and Its Effects on Offspring

GDM refers to a condition in which a nondiabetic woman develops hyperglycemia during pregnancy, resulting from insulin resistance caused by multiple pregnancy hormones and other factors. It increases the risk of miscarriage, premature delivery, intrauterine distress, fetal malformation, intrauterine death, intrauterine infection, macrosomia and hypertension during pregnancy, preeclampsia, and polyhydramnios [97].

In addition, women with GDM tend to have decreased serum VD levels, accompanied by upregulated mRNA expression of VDR and peroxisome proliferator-activated receptor gamma (PPAR-γ) in adipose tissue, which improves the progression of adipogenesis [98]. Additionally, for women with VD deficiency, several observational studies found that low VD levels are related to an increased risk of insulin resistance and GDM and emerging clinical evidence indicates that VD supplementation improves insulin sensitivity and glucose tolerance. A meta-analysis (nine cohort studies and six nested case-control studies with 40,788 participants and 1848 cases) reported that each 10 nmol/L increase in circulating 25(OH)D was associated with a 2% lower risk of GDM [99]. This is in line with the result in another meta-analysis, suggesting that individuals with VD deficiency had a 26% greater risk of developing GDM than those with normal serum 25(OH)D concentrations (OR: 1.3; 95% CI: 1.1~1.4) [100]. Moreover, a systematic review discovered that VD intervention during pregnancy could change the blood levels of VD, fasting plasma glucose, homeostasis model of assessment for insulin resistance index (HOMA-IR), glutathione, C-reactive protein (CRP), and lipid [101]. Moreover, to figure out whether the link of maternal VD deficiency to GDM is dependent on obesity in pregnant women, a cross-sectional study with 886 pregnant women conducted in Spain found a statistically significant prevalence ratio (PR) of 1.6 for GDM when VD deficiency was present, independent of mother’s BMI [102]. In a rat model, compared to the control group, pregnant rats with VD deficiency showed no significant difference in body weight, but a marked reduction in their offspring’s tissue sensitivity to exogenous insulin at 16 weeks [95]. Further study reported that the link between maternal VD deficiency and the risk of GDM is much greater among overweight/obese women in the Chinese population [103]. This outcome is in line with another study in China, which found that the protective effect of VD (VD > 20 ng/mL) for the development of GDM before 20 weeks of pregnancy is more significant in obese pregnant women (OR = 0.90) [104]. However, more longitudinal research on the potential effect of therapeutic VD supplementation to prevent GDM in pregnant women is still needed.

For offspring born to women with GDM, a recent meta-analysis highlighted that they are at a higher risk of being overweight with increasing age [105]. Additionally, studies have extensively found a relationship between GDM and the incidence of type 2 diabetes [106], cardiovascular disease [107], and cancer [108] in adulthood.

Furthermore, maternal VD deficit may exert potential impacts on the incidence of chronic disease of offspring later in life, in addition to the negative effects on bone mass and serum calcium concentrations among newborns. Maternal VD deficiency is related to increased insulin resistance among offspring in early childhood, as shown in a cohort study in America with 1882 mother–child pairs, revealing that a 10 nmol/L increase in maternal 25(OH)D was associated with a 0.007 (99%CI: −0.01, −0.001) nmol/L decrease in C-peptide, a 0.02 (99%CI: −0.03, −0.004) decrease in HOMA-IR, and a 0.13% (99%CI: –0.3, –0.003) decrease in body fat percentage at the age of 5~6 years old [109]. Another cohort study found that, at 9.5 years old, children of VD-deficient mothers (VD < 50 nmol/L) had higher fasting insulin resistance than did children of nondeficient women (*p* = 0.04). However, in this study, pre-pregnancy BMI of pregnant women with VD deficiency was unclear [110]. It has also been reported that maternal VD deficits are linked to cognitive disorders in offspring, including attention-deficit/hyperactivity disorder [111], schizophrenia [112], and learning disorders [113].

### 2.6. Clinical Evidence of VD Deficiency in Pregnant Women with Dyslipidemia, and Its Effect on Offspring

Dyslipidemia among pregnant women is associated with accelerated aging of the placenta and influences placental functions, including lipid storage. Moreover, premature placental aging may lead to adverse obstetric complications, such as preeclampsia, GDM, low birth weight and preterm birth [114]. Additionally, studies have shown that there are positive correlations between VD levels and total TC and triglyceride (TG) concentrations due to the physiological alterations of the responses to hormones in pregnant women, which indicates that maternal VD deficiency may be related to a proatherogenic metabolic status in pregnant women [115].

Among offspring born to mothers with dyslipidemia, increased birthweight, a higher risk of abnormal birth outcomes, such as macrosomia, largeness for gestational age, as well as atherosclerosis in adulthood were reported in several studies [116,117,118,119,120,121]. When pregnant women had VD deficiency, TG levels increased, and high-density lipoprotein cholesterol (HDL-C) levels decreased in the umbilical artery in a 425-participant study [122]. However, no associations of maternal 25(OH)D concentration with TG and HDL-C of offspring at four and six years of age were found [90]. Currently, clinical evidence on the relationship between maternal VD deficiency and offspring dyslipidemia needs further exploration.

### 2.7. Clinical Evidence of VD Deficiency in Pregnant Women with Other Disorders and Its Effect on Offspring

#### 2.7.1. Polycystic Ovary Syndrome (PCOS)

PCOS is a hormonal disorder among women of reproductive age. It induces higher blood glucose levels and higher blood pressure during pregnancy and increases the incidence of miscarriage or premature birth during pregnancy. For pregnant women with or without PCOS, 25(OH)D concentrations do not differ in them and their newborns [123]. However, considering the high prevalence of VD deficiency worldwide and the effects of VD on improving energy metabolism, lower VD levels among expectant mothers with PCOS exacerbate the symptoms of PCOS, such as insulin resistance, obesity, and hypertension, and elevate the risk of cardiovascular diseases and other chronic diseases in the long term [124].

In the offspring of women with PCOS, the cardiometabolic health of offspring was impacted by their mothers, with increased insulin resistance, altered lipid profile, and lower birthweight, especially in female offspring [125]. Presently, there is no research on the effects of VD status of pregnant women with PCOS on the metabolism of offspring.

#### 2.7.2. Metabolic Syndrome (MetS)

MetS is defined as an energy disorder characterized by central obesity, dyslipidemia, and hypertension and hyperglycemia. Studies have found that women with MetS are more likely to have pregnancy complications, such as preeclampsia and GDM, and cardiovascular disease and diabetes later in life [126]. Numerous studies have reported that VD insufficiency or deficiency increases the risk of MetS [127] and that VD supplementation improves outcomes in patients, including improving insulin sensitivity and β-cell function, decreasing fasting glucose level, insulin, and hemoglobin A1c (HbA1c), increasing HDL level and reducing LDL and TG, and reducing the incidence of type 2 diabetes [128]. As in pregnant individuals and their babies, a rat model suggested that maternal VD deficiency promotes MetS in both mothers and offspring, and VD treatment may attenuate the offspring’s MetS [129].

## 3. The Effects of Maternal VD Deficiency on Offspring Obesity, and Involved Mechanism

### 3.1. The Effects of Maternal VD Deficiency on the Adipogenesis Process in Offspring

Adipocytes are derived from mesenchymal stem cells (MSCs) obtained in adipose tissue. The progression of adipogenic differentiation includes two phases, namely the first phase, in which MSCs in the adipose vicinity commit to the preadipocyte lineage, and the second phase, in which they transform into adipocytes. The process involves many signaling molecules, and the two main regulators are PPAR-γ and C/EBP [130]. Activated PPAR-γ and C/EBPα upregulate the expression of genes involved adipogenesis and exert positive feedback on themselves and each other [131,132]. Many other regulators also play a part during this process, including sterol regulatory element-binding transcription factor 1c (SREBF1c), STAT5, and delta-like 1 (DLK1) [133,134,135]. In addition, there is evidence both in vivo and in vitro that VDR interacts with other regulators and influences the progression of adipogenesis, although these results are inconsistent [136,137,138].

In rat model, maternal VD deficiency before and during pregnancy is reported to promote the proliferation and differentiation of preadipocytes and adipocytes in male offspring and ultimately lead to an obese phenotype, including increased body weight and fat mass, in offspring of VD-deficient mothers. This may be related to the epigenetic alterations (changed methylation level of promoters and CpG islets) of certain genes. Compared to the control group, very low density lipoprotein receptor (*Vldlr*) gene was hypermethylated and expressed at low levels, and plays a key role in the metabolism of very low density lipoprotein. Additionally, hypoxia inducible factor 1 alpha subunit (*Hif1α*) gene was demethylated and highly expressed, bettering the adaptation to hypoxia by increasing oxygen delivery [42]. Such epigenetic alternations are associated with an increased risk of obesity in later life among offspring.

In another mouse model, Belenchia et al. [139] found that VD deficiency during pregnancy promoted the expression of adipogenic-regulating genes, PPAR-γ and VDR, in the perigonadal white adipose tissue of male offspring mice, though no significant differences in body weight were observed. In addition, maternal VD deficiency during pregnancy led to lower body weight among male offspring compared to the control group at weaning (three weeks of age; 9.9 ± 0.7 g vs. 10.4 ± 0.6 g, *p* = 0.01). However, within several weeks after weaning (4–7 weeks of age; Δ + 21.2 g vs. +19.1 g, *p* = 0.003), they underwent a rapid weight gain, and the expression of PPAR-γ in their liver was increased at the age of 19 weeks [140]. These results indicate that the effect of VD deficiency during pregnancy on the development of adipose tissue in offspring may be potentially long-term and may eventually make offspring predisposed to obesity more easily.

### 3.2. The Effect of Maternal VD Deficiency on Adipocytokine Secretion in Offspring

#### 3.2.1. Leptin

Leptin is a hormone secreted by adipose cells and other tissues, e.g., the fundic mucosa in stomach, to regulate energy balance and control body weight by curbing hunger. This peptide hormone also regulates fat storage and fetal development. In obese individuals, leptin resistance is characterized by high leptin levels, high levels of inflammation, and an uncontrollable food craving [141]. Previous human study reported leptin levels were negatively associated with VD levels in both genders [142,143]. Improper leptin levels in infants may pose a higher threat to obesity. In a pig model, a maternal VD-restricted diet caused offspring pigs to have higher fat mass (26.5 g/100 g vs. 23.2 g/100 g, *p* = 0.001), serum insulin (16.6 μmol/mL vs. 12.5 μmol/mL, *p* = 0.001), and leptin levels (1.6 ng/mL vs. 1.3 ng/mL, *p* = 0.01) [144]. In a human study, researchers provided VD supplement or placebo respectively for two groups of obese pregnant women. However, no correlations were found between maternal VD status and leptin level in cord blood (10.1 ng/dL vs. 9.6 ng/dL, *p* ≥ 0.001). This may because although different intervention of VD supplements or placebo led to significant differences between two groups in maternal 25(OH)D, the levels of maternal 25(OH)D of two groups were still above 75 nmol/L, a standard for VD sufficiency, at 24~28 weeks of gestation (120 nmol/L vs. 82 nmol/L, *p* < 0.001) and 35~37 weeks of gestation (123 nmol/L vs. 85 nmol/L, *p* < 0.001) [145]. Therefore, clinical evidence on the relation between maternal VD status and offspring leptin level remains scarce.

#### 3.2.2. Resistin

Resistin is another hormone made by white adipocytes. It accelerates the accumulation of low-density lipids in the liver and promotes inflammation and insulin resistance, leading to energy homeostasis impairment. In a clinical study, VD supplementation in obese adults increased their leptin (β[95%CI] = 22 [4,41], *p* = 0.02) and resistin levels (β[95%CI] = 14 [2,26], *p* = 0.02), and no significant changes were reported in nonobese individuals after VD supplementation [146]. However, to date, there has been limited human and animal evidence to explain the relationship between maternal VD status and offspring resistin levels.

#### 3.2.3. Adiponectin

Furthermore, adiponectin, an adipokine and a protein hormone secreted by adipocytes, regulates glucose levels and fatty acid metabolism. High molecular weight (HMW) adiponectin is the major bioactive isoform contributing [147]. In a multicenter study, researchers found that lower maternal VD levels in obese pregnant women made them more vulnerable to GDM and they had lower adiponectin, with a positive association between serum 25(OH)D concentrations and HMW-adiponectin level (r = 0.27, *p* = 0.007), indicating that adiponectin might serve as a predictor of GDM [148].

### 3.3. The Effect of Maternal VD Deficiency on Insulin Resistance in Offspring

Obesity is a triggering factor for insulin resistance and diabetes. The mechanisms of obesity-associated insulin resistance include abnormal lipid and glucose metabolism, inflammation, and oxidative response.

VD can modulate the biosynthesis of insulin and regulate the sensitivity of insulin by modulating gene expression or activating the PPAR-γ pathway. In a rat model, maternal VD restriction led to weight gain (+20%, *p* = 0.01) among their children and an increase in fat pad mass (+93%, *p* = 0.01) was observed even in grandchildren, accompanied by higher levels of insulin secretion, larger pancreatic islets, and lipid metabolism disorders as the offspring of VD-deficit mice showed marked hepatic steatosis and higher expression of fatty acid synthase in the liver [149]. Furthermore, in dams with VD deficiency, hepatic steatosis in offspring and downregulated PPAR-α in female offspring were found in several studies [149,150]. These findings suggest that lipid metabolism in the liver is significant in the effect of maternal VD deficiency on offspring insulin resistance. As for the pancreatic β-cell function to secrete insulin, a case-control study was conducted and suggested that maternal VD deficiency led to a lower level of the homeostasis model assessment of β-cell function (HOMA-β) (*p* = 0.01) compared to the control group with normal VD level, indicating a impaired function of secreting insulin [122]. In addition, another rodent study illustrated that a continuous increase in the level of inflammation played an important role in the development of insulin resistance in offspring whose mother rats were obese as HOMA-IR tended to be higher in the offspring of the group of VD-deficiency pregnant women than in those of the control group at 0, three, and eight weeks, and markedly higher at 16 weeks. Moreover, a glucose clamp test showed offspring of VD-deficient pregnant women had lower glucose infusion rates, indicating a lower insulin sensitivity [95]. In this study, the level of inhibitor of nuclear factor kappa-light-chain-enhancer of activated B cells α (*Iκbα*) gene methylation was increased in the liver of male offspring from VD deficient mothers, decreasing the expression of IκBα proteins. As regards IκBα protein inhibit inflammation, when this protein was downregulated, the levels of inflammatory factors, such as interleukin 6 (IL-6), IL-1β, IL-8, and tumor necrosis factor α (TNF-α), increased permanently.

### 3.4. The Effect of Maternal VD Deficiency on the Inflammatory Response in Offspring

Obese patients are constantly in a state of chronic, low-grade inflammation, exacerbating the development of obesity and insulin resistance. As lipids accumulate, the volume and number of fat cells increase. When fat cells are too large to rupture and undergo apoptosis, many macrophages are recruited and produce a variety of proinflammatory factors and chemokines, including CRP, TNF-α, IL-6, and IL-1β, etc., further enhancing the inflammatory response, eventually resulting in obesity-associated chronic inflammation. During this process, macrophages also promote preadipocyte hyperplasia by secreting pro-inflammatory factors, inducing insulin resistance and other metabolic disorders [151].

During pregnancy, maternal VD restriction in rats exacerbated the development of obesity in male offspring by increasing proinflammatory cytokines and decreasing anti-inflammatory cytokines, moderating immune cell populations and causing a polarization in the adipose tissue [152]. Apart from regulating immune cells, VD can also improve offspring inflammation levels through the regulation of some molecular signaling pathways. VD could inhibit signaling pathways related to angiogenesis, including insulin-like growth factor-1 receptor (IGF-1R), fibroblast growth factor (FGF), vascular endothelial growth factor (VEGF), and cell cycle regulation processes. It can also upregulate inflammatory response-related signaling pathways, such as Janus kinase (JAK)/signal transducer and activator of transcription (STAT) signaling, which is associated with macrophage development [153].

### 3.5. The Effect of Maternal VD Deficiency on Alternations of Gut Microbiota in Offspring

Early life is an important period for the evolution of the gut microbiota. During pregnancy, any change in the gut microbiome has been shown to make infants more susceptible to obesity, diabetes, asthma, and other chronic diseases in later life [154]. There are a range of factors that can affect gut microbiota development in early infancy, including antibiotic use during pregnancy, delivery mode (cesarean section or vaginal delivery), and the method of feeding (breast feeding or formula feeding) [155].

Studies have shown that an increase in maternal VD levels can help improve gut dysbiosis. Maternal VD supplementation was reported to increase Bacteroides (phyla) levels and reduce inflammatory responses in adult male offspring [156]. In addition to directly affecting the offspring intestinal microbiota, VD also plays an indirect role in affecting the metabolites of the intestinal microbiota.

Lipopolysaccharide (LPS) is a metabolite of gut microbiota related to the progression of obesity and insulin resistance, which can improve intestinal permeability, and increased serum LPS levels are associated with gut dysbiosis. Several animal models have demonstrated that male offspring of VD-deficient mothers have higher serum LPS levels, increased body inflammatory responses, decreased energy expenditure, increased blood sugar, and increased fat accumulation [157,158]. However, these alternations were not observed in female offspring [159].

### 3.6. The Effect of Maternal VD Status on Offspring Oxidative Stress

Obesity increases the level of oxidative stress with an increase in the level of ROS and a decrease in the level of antioxidant substances. Excessive ROS can lead to insulin resistance, lipid metabolism disorders, and abnormal adipokine secretion [160,161]. Experimental evidence also showed that in a gilt model, maternal obesity might increase oxidative stress in the placenta [162].

VD is known as a key factor in oxidative stress reduction and protection from oxidative stress-induced tissue impairments [163]. For instance, clinical evidence has demonstrated that among diabetic patients with hypertension, VD supplementation could improve vascular function [164]. Additionally, maternal VD deficiency inhibits placental development and leads to placenta dysfunction, causing fetal intrauterine growth restriction [165,166]. Currently, the mechanism of VD’s antioxidative effect is related to its mitochondrial oxidative phosphorylation capacity [49] as well as its induction of α-klotho proteins, an antiaging enzyme that promotes antioxidation by activating the molecular signaling pathway nuclear factor-erythroid factor 2-related factor 2 (Nrf2)/carbonyl reductase 1 (CBR1) [167]. As is illustrated in clinical evidence [168] and a rodent model [129], maternal VD deficiency might inhibit the expression of Nrf2 and CBR1 in the placenta, and therefore cause higher levels of oxidative stress, increasing the risk of metabolic disorders among offspring later in life.

## 4. Conclusions and Prospective

**Figure 1:**
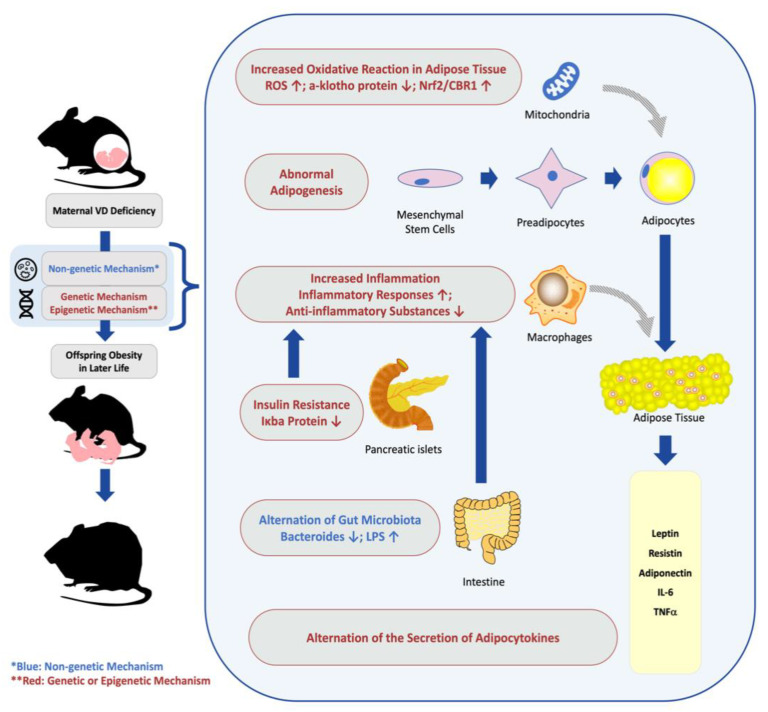
The mechanism on the effect of maternal VD deficiency on offspring obesity. Maternal VD deficiency increases vulnerability of offspring obesity through non-genetic mechanisms, genetic changes, and epigenetic alternations, such as increasing inflammation and oxidative reactions, affecting adipogenesis and adipocytokine secretion, and altering gut microbiota. VD Supplementation during pregnancy for women with VD deficiency may attenuate the negative metabolic outcomes in the offspring under medical guides. VD, vitamin D; IL-6, interleukin 6; TNF-α, tumor necrosis factor-α; ROS, reactive oxygen species; CBR1, carbonyl reductase 1; Nrf2, nuclear factor-erythroid factor 2-related factor 2; LPS, lipopolysaccharide; Iκbα, nuclear factor κB inhibitor α.

In conclusion, we summarized research on the relationships between maternal VD deficiency and offspring obesity in later life, and their potential mechanisms. The outcomes of studies on maternal VD deficiency and offspring obesity-related diseases are displayed in Table 1, and the potential mechanisms are shown in Table 2 and Figure 1. This conclusion suggests that VD plays a significant role in energy metabolism, and therefore may serve as a potential treatment or prevention of obesity in very early life. However, VD deficiency is still prevalent around the world, especially in the group of pregnant women and newborns. Thus, more public medical resources need to be invested to calculate and monitor the incidence of VD deficiency in different countries and areas, particularly in the vulnerable populations. Guidelines on VD deficiency exclusively for pregnant women are also needed to strengthen the awareness of this public health problem and clarify the procedure of VD deficiency treatment that adapts to the condition of different areas.

Recently, robust human and animal studies have demonstrated the potential mechanisms and alterations in fetuses and the placenta, which affect fetal growth and change energy metabolism in adulthood. Thus, it should not be neglected in regard to the discussion of the progression of obesity, compared with environmental factors existing in adulthood. However, thus far, although many studies have suggested that maternal VD deficiency influences offspring’s metabolic status and causes obesity in the long run, there are still findings contrary to this conclusion. This inconsistency may be the result of different experimental designs, the number of subjects, and other mixed factors. In addition, currently, plenty of studies have also proven the effects of maternal obesity on risk of offspring obesity, independent of maternal VD status [169,170,171,172,173,174], which suggests studies on the relation of maternal VD deficiency and offspring obesity should also consider pre-maternal BMI, the amount of energy attained from food, and the time spent on outdoor physical activities during gestation as confounding factors. Apart from maternal obesity, other metabolic disorders during pregnancy such as GDM also represent one of the confounding factors in the exploration of the effects of maternal VD deficiency on offspring obesity. Moreover, other potential confounding factors, such as maternal age, race/ethnicity, gestation weeks, season of serum sampling, paternal obesity, infant feeding style, breastfeeding duration, offspring age at measurement, offspring sex, offspring’s physical activity, maternal education, socioeconomic position, etc., should also be taken into consideration [175]. Thus, studies in the future need to separate this confounding factor before discussing how maternal VD deficiency affects offspring obesity. Moreover, further study on the mechanism and effects of maternal VD deficiency on offspring obesity is needed.

However, there are still some limitations to note. Due to the limited clinical and animal studies on some mechanisms, certain mother–fetal effects are still not clearly understood, such as the roles of adipocytokines and adipose tissue browning in the development of offspring obesity. As for gut microbiota, current research on the maternal-offspring effect of VD deficiency in obesity is only at the phyla level, and further studies are needed to determine how the gut microbiota is involved in the effect of maternal VD deficiency on offspring obesity. Currently, quantified measures at the microgram level are still relatively burdensome because of possible confounding factors. The limited follow-up and small scale of human research must also be addressed in further studies. Several studies without adjusted confounders, e.g., maternal BMI during pregnancy, also make it unclear to what degree maternal VD deficiency influences obesity in offspring. Overall, there is still a need for further large-scale, long-term clinical studies and more detailed animal studies to demonstrate how maternal VD influences the development of offspring obesity.

In the process of learning how different maternal factors contribute to the development of obesity in later life, there remains much more to be explored. How do epigenetic effects participate in the effects of VD on energy metabolism homeostasis? What other contributing factors participate in this progress? What intervention can be conducted to attenuate the progression of obesity in newborns of VD-deficient mothers? What strategies are recommended for VD supplementation during pregnancy? To answer these questions, we must better understand VD’s roles in pregnancy, and utilize VD supplementation more safely and efficiently, it is meaningful to explore the mechanisms behind the effects of maternal VD deficiency on obesity in offspring in later life to reduce the occurrence of obesity worldwide.

## Figures and Tables

**Table 1:** Cohort studies on VD deficiency during pregnancy and obesity and its related diseases in offspring.

Author [References]	Country/ Region	Subjects	Pre-Pregnancy BMI (kg/m^2^)	Outcomes
Tint et al. [89]	Singapore	292	23.8 ± 5.1 (VD inadequacy group) 22.4 ± 4.6 (VD sufficiency group)	2 weeks old: birth weight N *; abdominal subcutaneous adipose tissue volume ↑
Daraki et al. [90]	Greek	532	26.1 ± 5.8 (low VD group) 24.5 ± 4.5 (high VD group)	4 years old: BMI ↑; waist circumference ↑; TL N; TG N; HDL-C N. 6 years old: BMI ↑; waist circumference ↑; fat percentage ↑; TL N; TG N; HDL-C N
Miliku et al. [91]	Netherlands	4903	22.7 (18.1 ~ 34.8)	6 years old: BMI N; fat percentage ↑; lean mass percentage ↓; TL N; TG N
Crozier et al. [92]	United Kingdom	977	24.3 (22.2 ~ 27.6)	At birth: fat mass ↓; 4 and 6 years old: fat mass ↑
Morales et al. [93]	Spain	2223	18.5 ~ 25.0	1 years old: BMI ↑; 4 years old: BMI N
Jiang et al. [94]	China	329	21.2 ± 2.9 (VD sufficiency group) 21.1 ± 3.5 (VD insufficiency group) 21.0 ± 3.2 (VD deficiency group)	At birth: risks of preterm birth, small for gestation age, and low birth weight N; 0 ~ 3 years old: weight N; length N; BMI N
Hrudey et al. [109]	Netherlands	1882	24.2 ± 4.9 (VD sufficiency group) 23.6 ± 4.3 (VD insufficiency group) 22.2 ± 2.9 (VD deficiency group)	5~6 years old: insulin resistance ↑; fat percentage ↑
Krishnaveni et al. [110]	India	568	NA	9.5 years old: insulin resistance ↑; muscle-grip strength N; arm-muscle area N
Chen et al. [122]	China	425	22.6 ± 3.2 (VD deficiency group) 22.4 ± 2.8 (control group)	newborn: TG ↑; HDL-C ↓; HOMA-β ↓

* Abbreviation: BMI, body mass index; HDL-C, high density lipoprotein cholesterol; HOMA-β, homeostasis model assessment of β-cell function; TL, total cholesterol; TG, triglyceride; NA, not available; N, non-significant; ↑, increased; ↓, decreased.

**Table 2:** Animal studies on potential mechanism of VD deficiency during pregnancy on offspring obesity.

Author [References]	Animal Model	Intervention	Phenotypic Changes	Potential Mechanism
Wen et al. [42]	Sprague-Dawley rats	maternal VD * deficient diet	10 weeks: weight ↑; 14 weeks: 24 h heat production ↑, peak blood glucose ↑, adipose tissue volume ↑, blood lipid ↑	increased proliferation rate and number of lipid droplets for pre-adipocytes; hypermethylation and low expression of *Vldlr* gene; demethylation and high expression of *Hif1α* gene
Belenchia et al. [139]	C57BL/6J mouse	maternal VD deficient diet	body weight N, adipose pad weight N, adipocyte size N	male offspring: expression of PPAR-γ and VDR ↑
Belenchia et al. [140]	C57BL/6J mouse	maternal VD deficient diet	at weaning: weight ↓; 4 weeks: weight ↑; 19 weeks: perigonadal adipose tissue ↑	male offspring: adipocyte hypertrophy ↑; expression of PPAR-γ ↑
Guo et al. [144]	pigs	maternal VD deficient diet	fat mass ↑; insulin ↑; leptin ↑	FASN mRNA level ↑; altered LIPE gene expression in different tissues
Nascimento et al. [149]	Swiss Webster mouse	maternal VD deficient diet	body weight ↑; insulin ↑; AUC ↑; islet diameter ↑; liver steatosis	FASN expression ↑
Sharma et al. [150]	Wistar rats	maternal VD deficient diet	VD ↓; TG ↑; liver steatosis	female offspring: PPAR-γ and UCP2 ↓, SREBP-1c, IL-6 and SOD-1 ↑; male offspring: UCP2 and SOD-1 ↓
Zhang et al. [95]	Sprague-Dawley rats	maternal VD deficient diet	16 weeks: insulin ↑; HOMA-IR ↑	serum and liver IL-1β, IL-6, IL-8 and TNF-α ↑; hepatic Iκbα mRNA and IκBα protein ↓
Li et al. [152]	C57BL/6J mouse	maternal VD deficient diet	weight ↑; adipose cells ↑; abnormal glucose and lipid metabolisms	serum IL-4, IL-10, interferon-γ and TNF-α ↑; adipose tissue dendritic cells, and CD4(+) and CD8(+) T cells ↑; percentages of M1 macrophages ↑, percentages of M2 macrophages ↓
Villa et al. [156]	C57BL/6J mouse	maternal VD deficient diet	improved bone strength and structure	male offspring: colonic Bacteroides improved; systemic inflammation ↓;
Ni et al. [157]	C57BL/6J mouse	injection with 50 μg/kg LPS once	20 weeks: weight ↑; fat percentage ↑; energy expenditure ↓	mTOR/PPAR-γ ↑; serum bile acids level ↓, serum unsaturated fatty acids androgens and prostaglandins ↓
Villa et al. [158,159]	C57BL/6J mouse	maternal VD supplementation	fasting glucose ↓; fat mass ↓ in male offspring but not female offspring	intestinal permeability↓; serum LPS ↓ in male offspring but not female offspring
Zhang et al. [129]	Sprague-Dawley rats	maternal VD deficient diet	TG ↑; fasting glucose ↑; insulin ↑; HDL-C ↓	ROS level ↑; Nrf2/CBR1 pathway ↑

* Abbreviation: AUC, the area under the curve in the oral glucose tolerance test; CBR1, carbonyl reductase 1; FASN, fatty acids synthase; *Hif1α*, hypoxia inducible factor 1 alpha subunit; HOMA- IR, homeostasis model assessment of insulin resistance; Iκbα, nuclear factor κB inhibitor α; IL, interleukin; LIPE, hormone-sensitive lipase; LPS, lipopolysaccharide; mTOR, multi-component mechanistic target of rapamycin complex 1; Nrf2, nuclear factor-erythroid factor 2-related factor 2; PPAR-γ, adipogenic-regulating genes peroxisome proliferator-activated receptor gamma; ROS, reactive oxygen species; SOD-1, superoxide dismutase 1; SREBP-1c, sterol regulatory element-binding protein 1c; TNF-α, tumor necrosis factor-alpha; UCP2, uncoupling protein 2; VDR, vitamin D receptor; VD, vitamin D; *Vldlr*, very low density lipoprotein receptor; N, non-significant; ↑, increased; ↓, decreased.

## Data Availability

Not applicable.
